# Carotid artery stiffness and cognitive performance among men living with and without HIV

**DOI:** 10.1093/gerona/glag220

**Published:** 2026-09-03

**Authors:** Raymond Jones, Jessica Blair, Zenoria L Causey-Pruitt, Michael J Hankes, McKenna A Tharpe-Carter, David E Vance, Amanda L Willig, Daniel M Huck, Wendy S Post, Mary Clare Masters, Frank J Palella, Matthew J Mimiaga, Mallory D Witt, Jamie Peven, Sabina Haberlen, Mariam Aziz, Roland J Thorpe, Jr, Emily B Levitan, Thomas W Buford

**Affiliations:** Department of Medicine, University of Alabama at Birmingham, Birmingham, Alabama, United States; School of Public Health, University of Alabama at Birmingham, Birmingham, Alabama, United States; Department of Medicine, University of Alabama at Birmingham, Birmingham, Alabama, United States; School of Health Professions, University of Alabama at Birmingham, Birmingham, Alabama, United States; School of Health Professions, University of Alabama at Birmingham, Birmingham, Alabama, United States; School of Nursing, University of Alabama at Birmingham, Birmingham, Alabama, United States; TW Education, Dallas, Texas, United States; Department of Medicine, Brigham and Women’s Hospital, Harvard Medical School, Boston, Massachusetts, United States; Department of Medicine, Johns Hopkins University School of Medicine, Baltimore, Maryland, United States; Department of Epidemiology, Johns Hopkins University Bloomberg School of Public Health, Baltimore, Maryland, United States; Department of Medicine, Albert Einstein College of Medicine, Bronx, New York, United States; Department of Medicine, Northwestern University, Chicago, Illinois, United States; Department of Epidemiology, University of California Los Angeles, Los Angeles, California, United States; Department of Medicine, Lundquist Institute at Harbor—UCLA, Torrance, California, United States; Department of Psychiatry, University of Pittsburgh, Pittsburgh, Pennsylvania, United States; Department of Epidemiology, Johns Hopkins University Bloomberg School of Public Health, Baltimore, Maryland, United States; Department of Internal Medicine, Rush University Medical Center, Chicago, Illinois, United States; Department of Health, Behavior, and Society, Johns Hopkins University Bloomberg School of Public Health, Baltimore, Maryland, United States; School of Public Health, University of Alabama at Birmingham, Birmingham, Alabama, United States; Department of Medicine, University of Alabama at Birmingham, Birmingham, Alabama, United States; Birmingham/Atlanta VA Geriatric Research Education and Clinical Center, Birmingham, Alabama, United States; (Biological Sciences Section)

**Keywords:** Carotid distensibility, HIV, Cognitive function, Arterial stiffness, Vascular aging

## Abstract

Carotid artery stiffness has been associated with cognitive performance; however, the association among men with HIV (MWH) remains unclear. We examined the relationship between carotid artery distensibility and cognitive performance in the Multicenter AIDS Cohort Study (MACS). We analyzed data from 717 men (459 MWH and 258 men without HIV [MWoH]) who underwent carotid artery ultrasound and completed neuropsychological testing at least twice. Carotid artery distensibility was divided into tertiles. Cognitive outcomes included psychomotor speed (Trail Making Test A), executive function (Trail Making Test B), and processing speed (Symbol Digit Modalities Test). Multivariable mixed effects linear regressions were used to estimate longitudinal associations between distensibility tertiles and cognitive scores, with adjustments for socio-demographics, cardiovascular risk factors, and HIV-related characteristics. Regardless of HIV serostatus, lower vascular distensibility was associated with older age, higher systolic and diastolic blood pressure, and greater prevalence of cardiometabolic risk factors. In fully adjusted analyses, lower carotid artery distensibility was significantly associated with slower performance on Trail Making Tests A and B in MWH. These associations were weaker and not statistically significant among MWoH perhaps due to low sample size. In the combined sample, the rate of decline in executive function with age was similar across distensibility tertiles, but absolute scores were lowest in the low distensibility group. Lower carotid artery distensibility is linked to worse cognitive performance among MWH, suggesting that vascular aging may compound HIV-related cognitive decline.

## Introduction

Vascular risk factors have emerged as critical risk factors for cognitive decline among individuals living with HIV, who experience a 20%-60% prevalence of cognitive disorders.^1^ HIV-associated cognitive decline continues to pose significant challenges despite advancements in viral suppression achieved with potent combination antiretroviral therapy (ART)^2^ and has multifactorial pathogenesis. In this context, examining the role of vascular health markers, such as carotid artery stiffness, becomes important for identifying populations at risk for cognitive decline and for implementing targeted interventions.

Arterial stiffness, often assessed by measures such as carotid-femoral pulse wave velocity, reflects arterial wall integrity and elasticity, serving as a determinant of vascular aging and an independent predictor of atherosclerosis and cerebrovascular disease.^3^ Arterial stiffness is accelerated and accentuated by chronic conditions, such as HIV, hypertension, diabetes, and other comorbidities.^4–7^ The increase in stiffness of central arteries (branches that arise from the Circle of Willis and major cerebral arteries)^8^ and carotid arteries may lead to higher pressures, exposing distal end organs, such as the brain, to amplified pulsatile pressure oscillations.^3^^,^^4^^,^^7^^,^^9^ While the impact of HIV on cognitive functioning has been extensively studied, the specific role of arterial stiffness remains less clear. Previous evidence suggests an increase in arterial stiffness is associated with faster decline in executive functioning, information processing, and psychomotor speed in women living with HIV.^6^ Other evidence highlights increases in arterial stiffness resulted in declines in working memory and learning domains of cognitive performance in older people living with HIV.^4^ Associations exist between vascular pathology and cognitive decline in the general population and people living with HIV, while limited work compares these relationships, specifically, among men with HIV (MWH) and without HIV (MWoH). We focused on this population as sex differences are well documented in vascular structure and function and cognitive aging trajectories, which may cover some mechanistic associations. Specifically, in older adults, sex differences in the associations between vascular aging and its cognitive consequences are more pronounced in men compared with women.^10^^,^^11^ Additionally, MWH tend to have greater arterial stiffness and carotid plaque burden.^12^ Lastly, prior carotid stiffness and cognition work in women living with HIV already exists,^6^ which make this population of MWH understudied. Additionally, men represent the majority of adults living with HIV in the United States and bear a disproportionate burden of vascular risk factors that may accelerate cognitive aging. This study builds upon these aforementioned findings^4^^,^^6^^,^^7^ by highlighting the intertwined relationship between vascular health and cognitive performance, specifically, among MWH.

Leveraging data from the Multicenter AIDS Cohort Study (MACS), a longitudinal study of MWH and MWoH who have sex with men in the United States, we aimed to determine the associations of carotid artery stiffness on cognitive decline in MWH and MWoH. Utilizing a well-characterized cohort with comprehensive longitudinal clinical and neuropsychological assessments, we hypothesized that greater carotid artery stiffness at baseline would be associated with greater cognitive decline in both MWH and MWoH with the greatest change occurring in MWH.

## Methods

### MACS cohort

The MACS, now part of the MACS/Women’s Interagency HIV Combined Cohort Study (MWCCS), began in 1984 and enrolled men with or at risk of HIV in Baltimore/Washington DC, Pittsburgh, Chicago, and Los Angeles. Periods of MACS enrollment occurred in 1984-1985, 1987-1991, 2001-2003, and 2010-2017. Study details are published elsewhere.^13^ Briefly, MWH and MWoH attended semiannual visits (every 6 months) that included standardized interviews and demographic, clinical, laboratory assessments, and computer-assisted questionnaires that collect data on sexual behavior, drug and alcohol abuse, medical history, medications, depression, and quality of life.^13^^,^^14^ Blood was collected at each visit and used to test HIV antibodies, liver and kidney function, cholesterol and triglycerides, complete blood count, viral load (among MWH). Additionally, the participants were given physical exams. Approximately every 6 months, data were submitted to the MACS coordinating center.^13^^,^^15^

### Study population

We included participants in the preexisting Carotid Ultrasound substudy in the MACS (2004-2013) who also participated in the MACS neuropsychological assessment during the same period with at least 2 neurological exams. Eligibility in the preexisting substudy included age of at least 40 years old, weighed less than 300 pounds, and had no history of coronary heart disease (angina, myocardial infarction, or coronary revascularization).^16^

Sites received institutional review board approval and participants provided written informed consent. This analysis received approval by the MWCCS executive committee. Data necessary to replicate these analyses are available from MWCCS (https://statepi.jhsph.edu/mwccs/work-with-us/, mwccs@jhu.edu).

### Carotid cardiovascular measures

High-resolution B-mode ultrasound was used to image the right common carotid artery, internal carotid artery, and carotid bulb as previously described.^17^ The ultrasound scans were interpreted at a central location (University of Southern California Atherosclerosis Research Unit Core Imaging and Reading Center). The baseline carotid artery stiffness, assessed via carotid distensibility,^3^ was defined as the closest visit within a year of the baseline neuropsychological assessment visit. If multiple measurements were taken in this time period, we selected the measurement closest in time to the baseline neuropsychological assessment visit.

The ultrasound assessment included measurements of the right common carotid artery diameter at systole (RCCA_max_) and diastole (RCCA_min_), and pulse pressure was calculated from the mean of 5 brachial artery blood pressure measurements. These measures were included into the following formula to calculate distensibility:


$$\frac{2({\mathrm{RCCA}}_{\max}-{\mathrm{RCCA}}_{\min})/{\mathrm{RCCA}}_{\min}}{\mathrm{Pulse}\;\mathrm{Pressure}}.$$

Using this formula, distensibility was measured in units 10^−6^ × Newtons^−1^ × m^2^, where the lower values are indicative of a stiffer carotid artery.

### Neuropsychological outcomes

The neuropsychological testing in the MACS has been summarized elsewhere.^18^^,^^19^ Briefly, the MACS Neuropsychological Substudy was begun in 1988 following the reports of cognitive impacts of HIV. Beginning in 1988, the MACS semiannual (every 6 months) clinical assessment included the Trail Making Test A (TRLA; psychomotor speed), Trail Making Test B (TRLB; executive function), and the Symbol Digit Modalities Test (SDMT; speed of information processing). Tests were scored based on time to completion for Trail Making Test A and Trail Making Test B, and total correct for SDMT. Testing was performed by trained psychometrists using standardized protocols.^20^ The primary outcome of this study was longitudinal changes in score over time, operationalized as the rate of change (slope) in standardized cognitive scores over follow-up, for the 3 neuropsychological assessments available during the study period of 2004-2016. Standardized *Z*-scores were calculated as the difference between the individual test score and the normative sample mean score at baseline. Normative data were derived from MWoH, as previously done in prior studies in the MACS.^18^^,^^21^

### Covariates

We included self-reported demographics on age (years), race/ethnicity, education, and individual gross income (<$30 000/year). Participants were of Hispanic origin if they answered “yes” to the question “Are you of Hispanic (Spanish) or Latino origin?” Race was self-reported as White, Black, Asian, Pacific Islander, American Indian/Alaskan, and other. Due to limited sample size of certain groups, we categorized race/ethnicity as Hispanic (of any race), non-Hispanic Black, non-Hispanic White, and non-Hispanic Other. Education was based on highest education attained and was categorized as less than high school, high school graduate, some college, college graduate, or graduate school. Behavioral risk factors were self-reported smoking status (never, former, current) and alcohol use. Alcohol use was categorized as the following: none, low-moderate (1-2 drinks/day or 3-4 drinks/day for no more than once a month), moderate-heavy (3-4 drinks/day for more than once a month or ≥5 drinks/day for less than once a month), and binge (≥5 drinks/day for at least once a month).

Cardiometabolic risk factors were collected once a year during in-person visits via laboratory blood collections and physical examinations. These included body mass index (BMI; kg/m^2^), average systolic and diastolic blood pressure (mmHg) from 2 readings, self-reported anti-hypertensive medication usage, lipid assessment, self-reported cholesterol medication usage, and creatinine (mg/dL). In addition, we defined diabetes as HgA1c ≥6.5, glucose ≥126 (mg/dL), or previous diagnosis of diabetes with current use of anti-diabetic medication. Dyslipidemia was defined as fasting total cholesterol ≥200, low-density lipoprotein cholesterol ≥130, high-density lipoprotein cholesterol <40, triglycerides ≥150 (mg/dL), or use of lipid-lowering medication with self-report or clinical diagnosis. Lastly, the estimated glomerular filtration rate was based on the Chronic Kidney Disease Epidemiology Collaboration definition with race.^22^

HIV serostatus was assessed prospectively using enzyme-linked immunosorbent assay (ELISA) with Western blot confirmation. We analyzed HIV-related factors based on nadir CD4, current CD4 count, undetectable viral load (<50 copies/mL), duration of ART (years), type of ART, and history of AIDS/other opportunistic infections. Type of ART was categorized as none, protease inhibitor (PI) backbone in a combination regimen, non-nucleoside reverse transcriptase inhibitor (NNRTI)-based combination regimen without PI, NRTIs combination regimen without PI or NNRTI, or other regimen.

### Statistical analysis

Baseline characteristics were shown for the study sample size and stratified by HIV serostatus and age group (<50 vs. ≥50 years). The mean (± standard deviation) was shown for continuous variables while *N* (%) was shown for categorical variables. Statistical significance at α < 0.05 was represented using chi-square for categorical variables, or Fisher’s exact for small cell sizes, and equality of variances for continuous variables. In addition, baseline characteristics were tabulated by tertiles of carotid artery distensibility (low, middle, and high). These tables were also stratified by HIV serostatus and age group with statistical significance for continuous variables utilizing analysis of variance.

Multivariable linear mixed models with person-specific random intercepts and slopes were conducted to assess the association of baseline and repeated measures of vascular variables with the progression of cognitive decline over time. Time was operationalized as age in all models, and age was included as a random effect to capture heterogeneity in patterns of cognitive performance over time. Consistent with previous literature,^6^ fully adjusted models were utilized in analyses with the following variables: carotid distensibility, age, race/ethnicity, education, income, smoking status, alcohol use, BMI, blood pressure, anti-hypertensive medication usage, diabetes, total cholesterol, high-density lipoprotein, cholesterol medication usage, dyslipidemia, eGFR, creatinine, HIV serostatus, CD4 count, viral load, duration of ART, type of ART, AIDS diagnosis, and interactions. For analytical purposes, ART was categorized by regimen class rather than individual agents, consistent with prior literature in HIV and to preserve statistical power.^6^^,^^23^ To model HIV-specific characteristics, which are undefined for MWoH, we included interaction terms between HIV serostatus and the characteristics, without including the main effects of the characteristics. If the following interactions were significant at α < 0.05, then they were included in the models: carotid distensibility*age, carotid distensibility*HIV, carotid distensibility*age*HIV.

Multiple imputation was used for covariates and outcomes that were missing in analyses. All analyses were conducted using SAS, version 9.4 (SAS Institute, Cary, NC).

## Results

### Baseline characteristics

**Figure 1:**
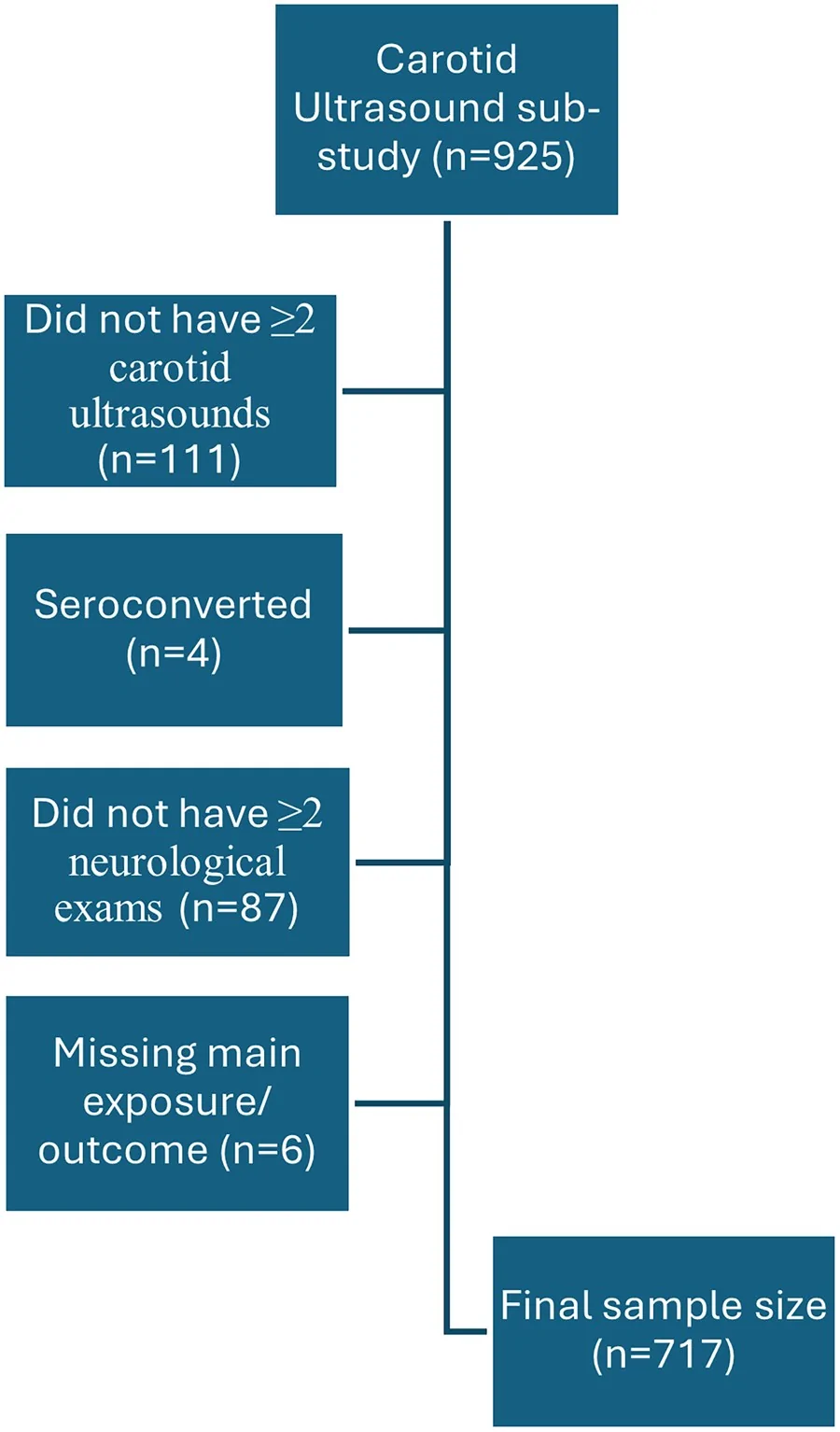
Study inclusion and exclusion flow diagram.

Of the eligible participants attending at least 2 visits during the study period that included the carotid ultrasound, 717 MACS participants had at least 2 neuropsychological assessments within 1.5 years of the baseline carotid examination. Participants who had not undergone a carotid ultrasound at least twice or did not have at least 2 neurological assessments were excluded from analyses (Figure 1). The time between the baseline carotid artery stiffness assessment and the neuropsychological assessment on average was 1.17 years (SD: 0.29) and ranged from 0 to 1.5 years.

Men in the study sample had a mean baseline age of 50 ± 7 years (Table 1). Most of the participants were non-Hispanic White (63%) or Black (29%). Compared with MWoH, MWH were younger (48.5 vs. 52.3 years; *p* < .001), had higher prevalence of less than high school education (7.8% vs. 3.9%; *p* < .001), income less than $30 0000 per year (48.4% vs. 35.6%; *p* < .001), and higher prevalence of current smokers (34.7 vs. 22.5; *p*-value: .003). Across the lower carotid distensibility tertile, indicative of higher arterial stiffness, overall MWH and MWoH were older (51.6 vs. 49.5 vs. 48.5 years; *p*-value: <.001) with higher BMI (26.6 vs. 26.0 vs. 25.1 kg/m^2^; *p*-value: .002), and higher systolic (128.2 vs. 124.6 vs. 118.7 mmHg; *p*-value: <.001) and diastolic (76.8 vs. 75.0 vs. 72.4 mmHg; *p*-value: <.001) blood pressure compared to middle and high carotid distensibility tertiles, respectively. Additionally, people in the lower distensibility tertile had a greater frequency of hypertension medication use (29.8% vs. 24.4% vs. 14.1%; *p*-value: <.001) compared to middle and high carotid distensibility tertiles. In addition, men in the low and middle tertiles were predominantly living with HIV. Among MWH, those with lower carotid distensibility had a higher use of NNRTI (38.5%) compared to men in the high carotid distensibility tertile who had a higher use of PIs (39.4%). MWH in the lower distensibility tertile had a significantly higher nadir CD4 T-cell count (333.0 vs. 278.9 cells/mm^3^; *p*-value: .04). At baseline, MWH and MWoH performed similarly on neuropsychological assessments. Similarly, in both MWH and MWoH, there were no significant differences in performance of neuropsychological assessments in the low tertile when compared with the middle and high tertile groups. Data shown in Table S1.

**Table 1:** Baseline characteristics of Multicenter AIDS Cohort Study population by HIV serostatus and carotid artery distensibility tertile, 2004-2006.

Carotid artery distensibility tertile								
	MWH (*n* = 459)				MWoH (*n* = 258)			
	Low (*n* = 144)	Middle (*n* = 178)	High (*n* = 137)	*p*-Value^a^	Low (*n* = 98)	Middle (*n* = 77)	High (*n* = 83)	*p*-Value^a^
Distensibility cut points	≤13.27	13.28–18.19	≥18.20		≤13.27	13.28–18.19	≥18.20	
**Vascular function**								
Carotid artery distensibility (10^−6^ × N^−1^ × m^2^)	9.6 (2.6)	15.7 (1.4)	23.6 (5.2)	—	10.0 (2.4)	15.6 (1.5)	22.9 (3.9)	—
**Cognitive scores**								
Trail Making Test A, seconds	22.3 (7.6)	21.2 (8.9)	21.5 (7.4)	.47	22.3 (8.7)	23.7 (8.7)	21.4 (7.4)	.21
Trail Making Test B, seconds	55.0 (29.7)	51.6 (28.3)	50.6 (29.1)	.40	52.7 (25.1)	52.8 (26.1)	47.4 (19.9)	.24
Symbol Digit Modalities Test, total correct	51.7 (13.0)	53.4 (13.9)	52.7 (11.6)	.49	53.7 (12.4)	52.2 (12.5)	56.3 (15.4)	.15
**Demographics**								
Age, years	49.9 (6.0)	48.4 (5.9)	47.2 (5.5)	**<.001**	54.0 (7.8)	51.9 (8.2)	50.6 (7.2)	**.01**
Race/ethnicity				**<.001^b^**				.41^b^
Non-Hispanic Black	61 (42.4)	56 (31.5)	31 (22.6)	—	21 (21.4)	21 (27.3)	14 (16.9)	—
Non-Hispanic White	73 (50.7)	112 (62.9)	81 (59.1)	—	72 (73.5)	52 (67.5)	61 (73.5)	—
Hispanic	7 (4.9)	8 (4.5)	24 (17.5)	—	5 (5.1)	4 (5.2)	8 (9.6)	—
Other	3 (2.1)	2 (1.1)	1 (0.7)	—	0 (0.0)	0 (0.0)	0 (0.0)	—
Education				**.03^b^**				.94^b^
<High school	11 (7.6)	9 (5.1)	16 (11.7)	—	4 (4.1)	4 (5.2)	2 (2.4)	—
High school graduate	18 (12.5)	34 (19.1)	12 (8.8)	—	10 (10.2)	8 (10.4)	11 (13.3)	—
Some college	52 (36.1)	47 (26.4)	41 (29.9)	—	21 (21.4)	12 (15.6)	16 (19.3)	—
College graduate	32 (22.2)	36 (20.2)	37 (27.0)	—	25 (25.5)	21 (27.3)	25 (30.1)	—
Beyond college graduate	31 (21.5)	52 (29.2)	31 (22.6)	—	38 (38.8)	32 (41.6)	29 (34.9)	—
Income, <$30 000/year	73 (50.7)	79 (44.4)	70 (51.1)	.40	33 (34.4)	32 (41.6)	26 (31.3)	.38
**Behavioral risk factors**								
Smoking status				.22^b^				.57^b^
Never	41 (28.7)	52 (29.6)	34 (24.8)	—	30 (30.6)	29 (37.7)	24 (28.9)	—
Former	44 (30.8)	71 (40.3)	56 (40.9)	—	46 (46.9)	29 (37.7)	42 (50.6)	—
Current	58 (40.6)	53 (30.1)	47 (34.3)	—	22 (22.5)	19 (24.7)	17 (20.5)	—
Alcohol use				.07^b^				.98^b^
None	27 (18.9)	41 (23.3)	22 (16.1)	—	12 (12.2)	9 (11.7)	11 (13.3)	—
Low–moderate	81 (56.6)	96 (54.6)	94 (68.6)	—	64 (65.3)	52 (67.5)	57 (68.7)	—
Moderate–heavy	18 (12.6)	26 (14.8)	16 (11.7)	—	16 (16.3)	10 (13.0)	10 (12.1)	—
Binge	17 (11.9)	13 (7.4)	5 (3.7)	—	6 (6.1)	6 (7.8)	5 (6.0)	—
**Cardiometabolic risk factors**								
BMI, kg/m^2^	26.3 (5.1)	25.7 (4.0)	24.6 (3.2)	**.004**	27.0 (4.3)	26.5 (5.9)	25.8 (5.1)	.31
Systolic BP, mmHg	127.4 (13.5)	124.5 (11.2)	118.7 (11.6)	**<.001**	129.7 (12.4)	124.9 (12.2)	118.5 (10.8)	**<.001**
Diastolic BP, mmHg	77.0 (8.7)	74.9 (8.3)	72.5 (7.9)	**<.001**	76.4 (8.2)	75.1 (8.9)	72.4 (7.7)	**.004**
Total cholesterol, mg/dL	198.7 (47.0)	191.6 (41.0)	188.2 (45.0)	.13	204.0 (39.4)	197.1 (38.4)	198.6 (34.6)	.43
HDL cholesterol, mg/dL	46.9 (13.7)	44.9 (14.5)	44.4 (14.3)	.29	49.2 (11.5)	50.0 (13.8)	51.0 (13.7)	.65
eGFR, mL/min/1.73 m^2^	90.1 (18.0)	88.6 (17.7)	89.9 (18.2)	.71	81.9 (16.1)	85.1 (17.1)	83.8 (16.8)	.44
Creatinine, mg/dL	1.1 (0.2)	1.1 (0.2)	1.0 (0.2)	.68	1.1 (0.2)	1.1 (0.2)	1.1 (0.2)	.88
Hypertension medication usage	42 (29.2)	41 (23.2)	18 (13.1)	**.005**	30 (30.6)	21 (27.3)	13 (15.7)	.06
Cholesterol medication usage	41 (28.7)	55 (31.3)	30 (21.9)	.18	19 (19.4)	17 (22.1)	10 (12.1)	.23
Diabetes	17 (12.8)	20 (12.1)	9 (6.7)	.21	8 (9.0)	10 (13.3)	7 (9.2)	.61
Dyslipidemia	114 (83.2)	150 (87.7)	116 (85.3)	.53	75 (82.4)	56 (74.7)	50 (62.5)	**.01**
**HIV-related factors**								
CD4 count, cells/mm^3^	589.2 (305.5)	544.7 (272.9)	519.2 (254.5)	.10	—	—	—	—
Nadir CD4, cells/mm^3^	333.0 (204.1)	285.5 (203.1)	278.9 (184.9)	**.04**	—	—	—	—
Undetectable viral load	93 (64.6)	112 (62.9)	79 (57.7)	.46	—	—	—	—
Duration of ART, years	4.7 (3.2)	4.6 (3.1)	4.6 (2.9)	.90	—	—	—	—
Type of ART combination regimen				.74^b^	—	—	—	—
None	35 (24.5)	48 (27.3)	37 (27.0)	—	—	—	—	—
PI-based	46 (32.2)	60 (34.1)	54 (39.4)	—	—	—	—	—
NNRTI-based	55 (38.5)	62 (35.2)	42 (30.7)	—	—	—	—	—
NRTI-based	6 (4.2)	6 (3.4)	4 (2.9)		—	—	—	—
Other	1 (0.7)	0 (0.0)	0 (0.0)	—	—	—	—	—
History of AIDS/other opportunistic infections	14 (9.7)	34 (19.1)	19 (13.9)	.06	—	—	—	—

Continuous variables are reported as mean (SD); categorical variables are reported as *n* (%). Differences in continuous variables were assessed using one-way analysis of variance (sum of squares). Differences in categorical variables were assessed using chi-square tests or Fisher’s exact tests (marked with superscript “b”) when cell sizes were small. HIV-related factors are presented only for MWH.

Abbreviations: AIDS, acquired immunodeficiency syndrome; ART, antiretroviral therapy; BMI, body mass index; BP, blood pressure; CD4, CD4+ T lymphocyte count; eGFR, estimated glomerular filtration rate; HDL, high-density lipoprotein; MWH, men with HIV; MWoH, men without HIV; NNRTI, non-nucleoside reverse transcriptase inhibitor; NRTI, nucleoside reverse transcriptase inhibitor; PI, protease inhibitor.

a *p*-Values represent overall comparisons across distensibility tertiles (low, middle, and high). **Bold **values denote *p*-value <0.05.

When stratified by <50 years (*n* = 276 MWH) vs. ≥50 years (*n* = 183 MWH) (Table S2), there were no significant differences between the groups in performance on neuropsychological assessments. Across age strata, individuals in the low distensibility tertile had significantly higher BMI (26.6 vs. 26.0 vs. 25.1 kg/m^2^; *p*-value: .002), systolic (128.2 vs. 124.6 vs. 118.7 mmHg; *p*-value: <.001) and diastolic (76.8 vs. 75.0 vs. 72.4 mmHg; *p*-value: <.001) blood pressure, and nadir CD4 T-cell counts (333.0 vs. 285.5 vs. 278.9 cells/mm^3^; *p*-value:.04) when compared with middle and high distensibility tertiles, respectively. Additionally, among men ≥50 years, carotid distensibility was lower across all tertiles when compared with men <50 years, indicative of greater arterial stiffness with age.

### Longitudinal associations of carotid distensibility with neuropsychological outcomes

Serial neuropsychological outcomes were performed at a median (range) of 3 (2-3) visits over 1.4 (0-6) years. Neuropsychological testing was complete every 6 months during the participant visit. Data presented are adjusted analyses controlling for age, race/ethnicity, education, income, smoking status, alcohol use, BMI, BP, antihypertensive medication, diabetes, total cholesterol, HDL, cholesterol medication, dyslipidemia, eGFR, creatinine, HIV status, CD4 count, viral load, duration of ART, type of ART, AIDS, and interactions.

In the combined sample of both MWH and MWoH, there was no statistically significant difference in the rate of psychomotor speed (TRLA) decline across distensibility tertiles (Vascular Function*Age Interaction *p *= .07). However, when examining within-tertile trajectories, psychomotor speed declined significantly over time in the low (β = −0.03 per year, *p *< .001) and middle (β = −.01 per year, *p *= .01) distensibility tertiles, whereas slope in the high distensibility tertile was smaller and did not reach statistical significance (β = −0.01 per year, *p* = .06) (Table 2 and Figure S1). In the combined group, for executive function, as measured by TRLB, the rate of decline did not differ across distensibility tertiles (Vascular Function*Age Interaction: *p *= .73). Nevertheless, significant within-tertile declines were observed in all 3 tertiles: low (β = −0.03 per year, *p *< .001); middle (β = −0.03 per year, *p *= .01); high (β = −0.02 per year, *p *= .0008). In the combined sample, speed of information processing, as measured by SDMT, the rate of decline did not differ across distensibility tertiles (Vascular Function*Age Interaction *p *= .73). Within-tertile changes were not significantly different for any distensibility tertile for speed of information processing.

**Table 2:** Rates of cognitive change within carotid distensibility tertiles, overall and by subgroup.

	Low distensibility	*p*-Value	Middle distensibility	*p*-Value	High distensibility	*p*-Value
**Overall**						
Trail Making Test A	**−0.03 (0.01)**	**<.0001**	**−0.01 (0.01)**	**.01**	−0.01(0.01)	.06
Trail Making Test B	**−0.03 (0.01)**	**<.0001**	**−0.03 (0.01)**	**<.0001**	**−0.02 (0.01)**	**.0008**
Symbol Digit Modalities Test	−0.001 (0.01)	.87	−0.001 (0.01)	.87	−0.01 (0.01)	.36
**Men with HIV**						
Trail Making Test A	**−0.03 (0.01)**	**<.0001**	−0.01 (0.01)	.39	**−0.02 (0.01)**	**.02**
Trail Making Test B	**−0.02 (0.01)**	**.02**	−0.02 (0.01)	**.06**	**−0.02 (0.01)**	**.02**
Symbol Digit Modalities Test	0.001 (0.01)	.49	0.004 (0.01)	.55	0.002 (0.01)	.81
**Men without HIV**						
Trail Making Test A	−0.01 (0.01)	.12	**−0.02 (0.01)**	**.04**	−0.004 (0.01)	.65
Trail Making Test B	**−0.03 (0.01)**	**.003**	**−0.03 (0.01)**	**.0006**	−0.02 (0.01)	.06
Symbol Digit Modalities Test	0.001 (0.01)	.75	0.002 (0.01)	.77	−0.01 (0.01)	.57
**Men <50 years**						
Trail Making Test A	−0.01 (0.03)	.59	**0.06 (0.03)**	**.04**	0.03 (0.02)	.18
Trail Making Test B	0.02 (0.03)	.41	0.02 (0.03)	.55	0.02 (0.02)	.21
Symbol Digit Modalities Test	0.03 (0.03)	.26	**0.06 (0.03)**	**.04**	0.001 (0.02)	.94
**Men ≥50 years**						
Trail Making Test A	**−0.03 (0.01)**	**.0002**	**−0.03 (0.01)**	**.005**	**−0.03 (0.01)**	**.001**
Trail Making Test B	**−0.04 (0.01)**	**<.0001**	**−0.03 (0.01)**	**.0004**	**−0.04 (0.01)**	**.001**
Symbol Digit Modalities Test	−0.003 (0.01)	.75	−0.0003 (0.01)	.97	−0.01 (0.01)	.30

Data reported as β (SE). Values represent estimated change per year (β per year) in cognitive test score derived from linear mixed-effects models. Bold entries are significant at *p* < .05. Separate, fully adjusted models presented above. Covariates include: age, race/ethnicity, education, income, smoking status, alcohol use, body mass index, blood pressure, antihypertensive medication, diabetes, total cholesterol, high-density lipoprotein, cholesterol medication, dyslipidemia, estimated glomerular filtration rate, creatinine, HIV status, CD4 count, viral load, duration of antiretroviral therapy (ART), type of ART, acquired immunodeficiency syndrome, and interactions.

In MWH, there was a statistically significant difference in the TRLA decline across distensibility tertiles (Vascular Function*Age: *p *= .01). Specifically, when examining within-tertile trajectories, psychomotor speed declined significantly over time in the low (β = −0.03 per year, *p *< .001) and high (β = −0.02 per year, *p *= .02) distensibility tertiles. However, the slope in the middle tertile did not reach statistical significance (β = −0.01 per year, *p* = .39). For executive function, measured using TRLB, the rate of decline did not significantly differ across distensibility tertiles (Vascular Function*Age Interaction: *p* = .95). However, significant within-tertile declines were observed in the low (β = −.02 per year, *p *= .02) and high tertile (β = −0.02 per year, *p *= .02) distensibility tertiles, whereas slope in the middle tertile did not reach statistical significance (β = −0.01 per year, *p *= .06). The rate of decline for speed of information processing (SDMT), among MWH, was not statistically different across the tertiles for MWH (Vascular Function*Age Interaction: *p *= .92). Within-tertile changes were not significantly different for any distensibility tertile for speed of information processing.

In MWoH, there was no statistically significant difference in the rate of executive function (TRLB) decline across distensibility tertiles (Vascular Function*Age Interaction: *p *= .48). However, when examining within-tertile slopes, executive function declined significantly over time in the low tertile (β = −0.03 per year, *p *= .003) and middle tertile (β = −0.03 per year, *p *< .001). The slope within the high tertile group was not significant (β = −0.02 per year, *p *= .06). Psychomotor speed (TRLA) and SDMT did not significantly differ across distensibility tertiles. Within-tertile changes were also not significant among distensibility tertiles for both psychomotor speed TRLA and SDMT.

For men ≥50 years, there was no statistically significant difference in the TRLA decline across distensibility tertiles (Vascular Function*Age Interaction: *p *= .65). However, within-tertile trajectories indicated significant declines in the slope of low (β = −0.03 per year, *p *< .001), middle (β = −0.03 per year, *p *= .005), and high (β = −0.03 per year, *p *< .001) distensibility tertiles. Similarly, among men ≥50 years, there was no statistically significant difference in the TRLB decline across distensibility tertiles (Vascular Function*Age Interaction: *p *= .89). Within-tertile trajectories for executive function (TRLB) were similar to psychomotor speed (TRLA), such that there were significant declines in the slopes of all distensibility tertiles for executive function (TRLB): low tertile (β = −0.04 per year, *p *< .0001); middle tertile (β = −0.03 per year, *p *< .001); high tertile (β = −0.04 per year, *p *< .001). In men aged ≥50 years, SDMT did not significantly differ across distensibility tertiles. Within-tertile changes were also not significant among any distensibility tertile for SDMT.

In men <50 years, there was no statistically significant difference in the TRLA decline across distensibility tertiles (Vascular Function*Age Interaction: *p *= .17). Interestingly, when examining within-tertile trajectories, psychomotor speed (TRLB) significantly increased over time only in middle distensibility tertile (β = 0.06 per year, *p *= .04). In this group of men aged <50 years, there was no statistically significant difference in the TRLB decline across distensibility tertiles (Vascular Function*Age Interaction: *p *= .96). Within-tertile changes were not observed for executive function (TRLB) in any distensibility tertiles. Similar to TRLA, there was no statistically significant difference in the SDMT decline across distensibility tertiles (Vascular Function*Age Interaction: *p *= .24). Though, when examining within-tertile trajectories, SDMT significantly increased over time only in the middle distensibility tertile (β = 0.06 per year, *p *= .04).

Interactions of distensibility with HIV serostatus were significant only for psychomotor speed, suggesting that MWH status in the low distensibility tertile at baseline was associated with faster rate of decline in psychomotor speed measured by TRLA with increasing age (β = −0.03, *p *< .001) when compared with MWH in the highest distensibility tertile (β = −0.02, *p *= .02). The 3-way interaction of carotid distensibility*age*serostatus was not significant for any neuropsychological outcome.

## Discussion

### Overview

This study investigated the relationship between baseline carotid artery stiffness, measured via carotid distensibility, and cognitive performance among MWH and MWoH. Leveraging data from the MACS cohort, we sought to determine whether vascular aging contributes to cognitive deterioration, particularly in MWH. Our findings demonstrate that lower carotid distensibility, indicative of greater arterial stiffness, is associated with faster decline in psychomotor speed among MWH. A similar association was not observed in MWoH; however, formal interaction tests between vascular function and HIV serostatus were not significantly different, and analyses of the combined cohort demonstrated comparable overall patterns. Given the smaller sample size of MWoH, the apparent differences by serostatus should be interpreted cautiously. Rather than indicating definitive HIV-specific susceptibility, these results suggest that vascular aging may be an important contributor to cognitive decline, particularly in a population with higher vascular burden and warrant further investigation in larger studies designed to test effect modification by HIV status.

### Interpretation of findings

In our combined cohort of MWH and MWoH, we found that cognitive function declined with age across multiple domains, but the relationship between carotid distensibility and longitudinal change in cognitive functioning was domain- and group-specific. Executive function significantly declined over time across all tertiles of distensibility, yet the rates of decline were similar, suggesting that age rather than arterial stiffness was the primary driver of decline in this domain. In contrast, low distensibility was associated with a steeper decline in psychomotor speed, particularly among MWH. This indicates that advanced arterial stiffness in MWH may contribute to more rapid declines in speed and attention tasks. Processing speed demonstrated no meaningful changes over time in any distensibility group, reinforcing that not all cognitive domains are equally sensitive to vascular aging. Notably, men age ≥50 years experienced steeper declines in both psychomotor processing and executive function, while men aged <50 years showed stable or even improved performance over time, possibly because of practice effects. Collectively, these findings suggest that arterial stiffness may be more strongly related to psychomotor decline in MWH, although formal interaction tests did not reach statistical significance. However, executive function is more strongly related to aging independent of vascular stiffness. Several methodological factors may also contribute to the observed variability. Cognitive domains differ in measurement precision and longitudinal reliability, which may affect the ability to detect change over time.^24^^,^^25^ Selective survivorship and attrition in longstanding cohorts may further attenuate observed associations.^26^ Additionally, while models adjusted for known confounders, residual confounding and temporal misalignment between vascular and cognitive assessments must be considered.

### Comparison with previous literature

Prior studies have consistently demonstrated links between vascular health and cognitive function.^6^^,^^27^ Arterial stiffness has been associated with impaired executive function and psychomotor speed in aging populations.^3^ In people living with HIV, these associations may be amplified due to chronic inflammation^28^^,^^29^ and endothelial dysfunction.^6^ Our study builds on this literature by showing that carotid stiffness is associated with cognitive decline specifically in MWH. This finding complements Huck et al., who found that higher carotid stiffness was independently associated with faster decline in executive functioning and psychomotor speed among women with or at risk for HIV. Notably, their study focused on the predominantly female WIHS cohort, while ours addresses a critical gap by examining men.^6^ We previously reported associations between estimates of arterial stiffness and cognitive functioning in adults with HIV, reinforcing the vascular contributions to cognitive aging in this population.^4^ Importantly, as older age was associated with both greater arterial stiffness and worse cognitive performance, these relationships do not, alone, establish causal or mediating role for vascular aging. Age may independently influence both vascular and cognitive processes.^30–32^ Rather, our findings suggest that vascular stiffness may represent one of several age-related biological pathways contributing to cognitive change, particularly in domains related to processing speed.

As life-expectancy in people living with HIV has increased with the use of ART, so has cardiovascular disease prevalence.^33^ Specifically, HIV serostatus and use of ART is associated with an increased risk of arterial stiffness.^7^ Low CD4+ T-cell counts have also been found to be a major atherosclerosis risk factor in individuals living with HIV.^16^ However in the present study, MWH in the lowest distensibility tertile exhibited a higher nadir CD4 T-cell count. This result may suggest that immunosuppression alone may not fully account for arterial stiffening in MWH. While nadir CD4 may capture past disease severity, it does not reflect cumulative effects on the vasculature, duration on ART exposure, or age-related vascular remodeling which may contribute to arterial stiffness. Importantly, this finding may underscore that arterial stiffness may represent an independent pathway linking HIV to cognitive performance rather than a downstream impact of immunocompromise. Therefore, the observed findings reflect vascular mechanisms that may persist despite preserved immune indices highlighting arterial stiffness as a modifiable target supporting brain health in MWH.

Further, a study comparing ischemic stroke incidence in patients with and without HIV in a US healthcare system found that patients with HIV were at an increased risk of stroke.^34^ This elevated risk was especially pronounced among women and young patients and was independent of common stroke risk factors.^34^ Together, these findings suggest that vascular aging and HIV serostatus may share mechanisms underlying cognitive decline. By focusing specifically on men, this study extends existing literature showcasing that greater arterial stiffness is longitudinally associated with declines in psychomotor speed, which is a cognitive domain that is sensitive to vascular dysfunction and white matter pathology.^35^^,^^36^ Psychomotor speed tends to depend on white matter and frontal-subcortical integrity, which are vulnerable to poor cerebral perfusion and arterial remodeling.^37^^,^^38^ These findings provide longitudinal, cohort-based evidence supporting vascular pathways as 1 potential mechanism linking HIV, cardiovascular risk, and cognitive declines in aging men.

### Plausible mechanisms

Early stages of arterial stiffening can expose cerebral microvasculature to mechanical strain and small vessel injury. Previous evidence implicated chronic cerebral hypoperfusion and microvascular damage in slower processing speed and impaired psychomotor and executive functioning.^39–41^ In the context of HIV, persistent immune activation and chronic systemic inflammation may exacerbate vascular dysfunction, leading to premature arterial stiffness and cognitive decline.^7^^,^^29^ Traditional risk factors, which were more prevalent in our study among men with lower distensibility (eg, higher blood pressure, BMI, hypertension medication use, and smoking status), may interact with HIV-related vascular changes to further contribute to cognitive decline.^36^ These mechanisms may suggest a potential pathway in which HIV and vascular aging converge to selectively impair cognitive domains, though future work is needed to determine specific domains. Moreover, while ART improves cognitive performance in MWH, long-term use of some ART medications can further increase arterial stiffness and cardiovascular risk, possibly because of metabolic side effects related to dyslipidemia, glucose intolerance, and lipodystrophy.^5^^,^^7^^,^^29^^,^^42–44^ Although newer ART regimens may pose a lower risk, longitudinal data on modern medications are sparse.^45^

### Clinical and public health implications

Our findings underscore the importance of monitoring vascular health in HIV care. As MWH live longer due to effective ART,^46^ preserving cognitive function becomes increasingly relevant.^2^ By elucidating the role of specific vascular risk factors, our findings may have implications for targeted interventions aimed at preserving cognitive function and reducing the burden of cognitive disorders among MWH. Interventions targeting vascular health (eg, blood pressure control, lipid management, and lifestyle modifications) could promote positive cardiovascular and cognitive outcomes.^34^ Given the success of ART in extending life expectancy, the focus must now shift toward preserving quality of life, including cognitive and cardiovascular health.

### Strengths and limitations

Our study has several notable strengths. First, it involves a large cohort of both MWH and MWoH with repeated cognitive and vascular measurements, allowing for assessment of longitudinal trajectories rather than relying on a single, cross-sectional analysis. Second, the use of carotid distensibility as a direct measure of arterial stiffness provides an objective and reproducible marker of vascular aging. Lastly, the evaluation of both MWH and MWoH controls enabled the examination of the combined and separate effects of HIV serostatus, aging, and arterial stiffness on cognitive outcomes.

Several limitations should be acknowledged. First, carotid distensibility was measured only at baseline and therefore may not fully reflect longitudinal changes in arterial stiffness over the follow-up period of this study. However, the long duration of follow-up allowed for assessment of cumulative cognitive change over time and modest annual declines, particularly in psychomotor speed may be clinically meaningful when sustained over many years. Second, although we adjusted for key clinical and demographic variables, other unmeasured factors such as cerebrovascular disease, lifestyle behaviors, and genetic or epigenetic factors, may potentially confound our findings. We also observed improvements in some cognitive domains among younger men, suggesting possible practice effects in this study with relatively short assessment intervals, which could attenuate our results. Next, there was a smaller sample of MWoH, which limited statistical power to detect associations in this subgroup. This may explain why the relationship between arterial stiffness and psychomotor decline was observed among MWH but not MWoH. Finally, because this was an observational study, a causal link between arterial stiffness and cognitive decline cannot be determined. This study relied on older baseline data (2004-2006); thus, much HIV treatment and adherence protocol has since changed which may have an impact on current regimens. Lastly, the use of ART regimens varied over the time period that these data were collected, as new regimens were introduced with better efficacy at controlling viral replication and favorable side effect profiles.^47^^,^^48^ In the MACS study population, use of efavirenz/tenofovir disoproxil fumarate/emtricitabine (Atripla) increased from introduction until 2011, when 26% of the population used it and it was the most commonly used regimen, and then decreased thereafter. Dolutegravir/abacavir/lamivudine (Triumeq) only became commonly used in 2015,^48^ after the data used in this analysis were collected. While antiretroviral medications may influence cognition through changes in the vasculature,^36^ the effects of specific antiretroviral regimens on cognition and potential mechanisms are beyond the scope of this study.

### Future directions

These findings underscore the importance of early identification and intervention in populations with elevated cardiovascular risk, regardless of HIV serostatus. Routine vascular assessments, such as carotid ultrasound, may help detect individuals at risk for cognitive decline and guide preventive strategies. Our results support integrating cardiometabolic risk factor management (eg, blood pressure control, lipid regulation, and lifestyle interventions [eg, exercise, diet, etc.]) into cognitive health strategies for aging individuals with or at risk for HIV. Longer, interventional studies are also needed to investigate cognitive decline over time and evaluate the effectiveness of vascular interventions. As demonstrated by the MACS cohort’s long-term retention and data quality,^13^^,^^14^ such studies are feasible and essential for understanding aging in HIV populations.

## Conclusions

To conclude, in this longitudinal cohort of MWH and MWoH, we observed that lower carotid distensibility, a marker of greater arterial stiffness, was associated with faster decline in psychomotor speed, particularly among MWH. In contrast, the decline in executive function appeared to be primarily age-related and independent of arterial stiffness, and processing speed remained relatively stable across arterial stiffness tertiles. These findings suggest that vascular aging and HIV may contribute to accelerated age-related decline in psychomotor speed. Identifying and managing cardiovascular risk factors may mitigate cognitive decline in this population. Future research incorporating longitudinal vascular assessments and neuroimaging is warranted to determine causal pathways and to identify targets for prevention and intervention.

## Supplementary Material

glag220_Supplementary_Data

## Data Availability

The data that support the findings of this study are available upon request through the MWCCS consortium-established concept sheet submission process. For more information, visit https://statepi.jhsph.edu/mwccs/. Requests to access data can be made on the website.
