# Supplementary material for: Carotid artery stiffness and cognitive performance among men living with and without HIV
Source: J Gerontol A Biol Sci Med Sci. 2026 Sep 3;81(10):glag220. doi: 10.1093/gerona/glag220 (PMC13598940; doi:10.1093/gerona/glag220)

Title: Carotid Artery Stiffness and Cognitive Performance among Men Living with and without HIV
Supplemental Materials

Supplemental Table 1. Baseline characteristics of MACS study population by age group and distensibility tertile

	Carotid artery distensibility							
	<50 years (n=380)				≥50 years (n=337)			
	Low (n=103)	Middle (n=144)	High (n=133)	p-value	Low (n=139)	Middle (n=111)	High (n=87)	p-value
**Distensibility Cutpoints:**	≤13.27	13.28 – 18.19	≥18.20		≤13.27	13.28 – 18.19	≥18.20	
**Vascular function**								
Carotid artery distensibility ($10^{-6} \times N^{-1} \times m^2$)	10.5 (2.3)	15.7 (1.4)	24.0 (5.1)	—	9.3 (2.6)	15.6 (1.4)	22.3 (3.8)	—
**Cognitive scores**								
Trail Making Test A, seconds	21.2 (6.9)	22.5 (10.1)	21.5 (7.2)	0.40	23.2 (8.7)	21.3 (7.0)	21.3 (7.7)	0.11
Trail Making Test B, seconds	50.5 (22.4)	54.8 (31.9)	50.6 (24.7)	0.34	56.7 (31.1)	48.3 (20.4)	47.6 (28.0)	***0.02***
Symbol Digit Modalities Test, total correct	52.9 (12.7)	51.8 (13.6)	52.9 (12.7)	0.73	52.2 (12.9)	54.6 (13.2)	55.8 (14.0)	0.11
**Demographics**								
Age, years	44.9 (2.7)	44.7 (2.6)	44.1 (2.7)	—	56.5 (5.0)	55.7 (5.6)	55.1 (4.3)	—
Race/ethnicity				***<0.001^***				***0.003^***
Non-Hispanic Black	43 (41.8)	60 (41.7)	37 (27.8)	—	39 (28.1)	17 (15.3)	8 (9.2)	—
Non-Hispanic White	51 (49.5)	72 (50.0)	65 (48.9)	—	94 (67.6)	92 (82.9)	77 (88.5)	—
Hispanic	7 (6.8)	10 (6.9)	30 (22.6)	—	5 (3.6)	2 (1.8)	2 (2.3)	—
Other	2 (1.9)	2 (1.4)	1 (0.8)	—	1 (0.7)	0 (0.0)	0 (0.0)	—
Education				0.43^				0.18^
<High school	9 (8.7)	12 (8.3)	17 (12.8)	—	6 (4.3)	1 (0.9)	1 (1.2)	—
High school graduate	14 (13.6)	32 (22.2)	18 (13.5)	—	14 (10.1)	10 (9.0)	5 (5.8)	—
Some college	36 (35.0)	38 (26.4)	40 (30.1)	—	37 (26.6)	21 (18.9)	17 (19.5)	—
College graduate	28 (27.2)	34 (23.6)	36 (27.1)	—	29 (20.9)	23 (20.7)	26 (29.9)	—
Beyond college graduate	16 (15.5)	28 (19.4)	22 (16.5)	—	53 (38.1)	56 (50.5)	38 (43.7)	—
Income, <$30,000/year	51 (49.5)	78 (54.2)	72 (54.1)	0.72	55 (40.2)	33 (29.7)	24 (27.6)	0.09
**Behavioral risk factors**								
Smoking status				0.25^				0.18^
Never	35 (34.0)	42 (29.6)	34 (25.6)	—	36 (26.1)	39 (35.1)	24 (27.6)	—
Former	24 (23.3)	47 (33.1)	49 (36.8)	—	66 (47.8)	53 (47.8)	49 (56.3)	—
Current	44 (42.7)	53 (37.3)	50 (37.6)	—	36 (26.1)	19 (17.1)	14 (16.1)	—
Alcohol use				0.09^				0.21^
None	16 (15.5)	31 (21.8)	27 (20.3)	—	23 (16.7)	19 (17.1)	6 (6.9)	—
Low – moderate	57 (55.3)	78 (54.9)	88 (66.2)	—	88 (63.8)	70 (63.1)	63 (72.4)	—
Moderate – heavy	16 (15.5)	21 (14.8)	10 (7.5)	—	18 (13.0)	15 (13.5)	2 (2.3)	—
Binge	14 (13.6)	12 (8.5)	8 (6.0)	—	9 (6.5)	7 (6.3)		—

**Cardiometabolic risk factors**								
BMI, kg/m^2	26.6 (5.1)	26.4 (5.1)	24.8 (3.5)	***0.005***	26.6 (4.5)	25.4 (3.9)	25.4 (4.7)	0.07
Systolic BP, mmHg	126.9 (11.8)	125.9 (10.9)	119.0 (11.8)	***<0.001***	129.4 (14.0)	122.9 (12.0)	118.1 (10.5)	***<0.001***
Diastolic BP, mmHg	76.0 (8.3)	75.5 (8.7)	72.8 (8.6)	***0.007***	77.4 (8.6)	74.3 (8.2)	71.8 (6.5)	***<0.001***
Total cholesterol, mg/dL	192.5 (41.2)	190.1 (41.4)	189.2 (41.7)	0.82	207.2 (45.1)	197.5 (38.5)	196.6 (41.4)	0.10
HDL cholesterol, mg/dL	47.3 (14.0)	45.9 (15.4)	45.9 (13.4)	0.68	48.2 (11.9)	47.1 (13.3)	48.4 (15.8)	0.74
eGFR, mL/min/1.73 m^2	93.4 (17.0)	91.9 (17.6)	92.8 (17.5)	0.81	81.8 (16.6)	81.9 (15.8)	79.5 (15.4)	0.54
Creatinine, mg/dL	1.0 (0.2)	1.1 (0.2)	1.0 (0.2)	0.32	1.1 (0.2)	1.1 (0.2)	1.1 (0.2)	0.62
Hypertension medication usage	17 (16.5)	29 (20.3)	12 (9.0)	***0.03***	55 (39.6)	33 (29.7)	19 (21.8)	***0.02***
Cholesterol medication usage	21 (20.4)	32 (22.5)	16 (12.0)	0.06	39 (28.3)	40 (36.0)	24 (27.6)	0.32
Diabetes	7 (7.2)	17 (12.4)	6 (4.7)	0.07	18 (14.4)	13 (12.5)	10 (12.1)	0.86
Dyslipidemia	75 (76.5)	114 (82.6)	100 (76.9)	0.41	114 (87.7)	92 (85.2)	66 (76.7)	0.09
**HIV-related factors**								
MWH	71 (68.9)	110 (76.4)	95 (71.4)	0.40	73 (52.5)	68 (61.3)	42 (48.3)	0.16
CD4 count, cells/mm^3	597.6 (298.3)	516.6 (271.9)	498.0 (241.7)	0.05	580.7 (314.5)	589.4 (270.3)	567.0 (278.3)	0.93
Nadir CD4, cells/mm^3	361.4 (222.4)	303.8 (224.1)	281.5 (174.4)	0.05	305.8 (182.2)	256.4 (162.0)	273.4 (207.9)	0.27
Undetectable viral load	47 (66.2)	64 (58.2)	50 (52.6)	0.21	46 (63.0)	48 (70.6)	29 (69.1)	0.61
Duration of ART, years	4.9 (3.3)	4.4 (3.1)	4.4 (2.9)	0.51	4.6 (3.2)	4.9 (3.1)	5.1 (3.0)	0.67
Type of ART				0.35^				0.98^
None	16 (22.5)	31 (28.7)	27 (28.4)	—	19 (26.4)	17 (25.0)	10 (23.8)	—
PI	22 (31.0)	35 (32.4)	40 (42.1)	—	24 (33.3)	25 (36.8)	14 (33.3)	—
NNRTI	28 (39.4)	37 (34.3)	26 (27.4)	—	27 (37.5)	25 (36.8)	16 (38.1)	—
NRTI	4 (5.6)	5 (4.6)	2 (2.1)	—	2 (2.8)	1 (1.5)	2 (4.8)	—
Other	1 (1.4)	0 (0.0)	0 (0.0)	—	0 (0.0)	0 (0.0)	0 (0.0)	—
History of AIDS/other opportunistic infections	7 (9.9)	19 (17.3)	11 (11.6)	0.29	7 (9.6)	15 (22.1)	8 (19.1)	0.12

Continuous variables are reported as mean (SD); categorical variables are reported as n (%). Differences in continuous variables were assessed using one-way ANOVA (sum of squares). Differences in categorical variables were assessed using Chi-square tests or Fisher's exact tests (marked with ^) when cell sizes were small. HIV-related factors are presented only for men with HIV (MWH). Abbreviations include: MWH, men with HIV; MWoH, men without HIV; ART, antiretroviral therapy; PI, protease inhibitor; NNRTI, non-nucleoside reverse transcriptase inhibitor; NRTI, nucleoside reverse transcriptase inhibitor; BMI, body mass index; BP, blood pressure; HDL, high-density lipoprotein; eGFR, estimated glomerular filtration rate; CD4, CD4+ T lymphocyte count; AIDS, acquired immunodeficiency syndrome

Supplemental Table 2. Baseline Characteristics of MACS Study Population: Overall, Serostatus, and Age Strata

		HIV Serostatus			Age group		
**Characteristic**	**Overall (n=717)**	**MWH (n=459)**	**MWoH (n=258)**	***p*-value**	**<50 (n=380)**	**≥50 (n=337)**	***p*-value**
Vascular function							
Carotid artery distensibility	16.0 ± 6.3	16.2 ± 6.4	15.8 ± 6.1	0.47	17.2 ± 6.4	14.7 ± 5.9	**<0.001**
Cognitive scores							
Trailmaking Test A, seconds	21.9 ± 8.2	21.6 ± 8.1	22.4 ± 8.3	0.21	52.5 ± 13.0	53.9 ± 13.3	0.15
Trailmaking Test B, seconds	51.9 ± 27.3	52.4 ± 29.0	51.0 ± 23.9	0.51	52.1 ± 27.1	51.6 ± 27.4	0.79
Symbol Digit Modalities Test, total correct	53.1 ± 13.2	52.6 ± 13.0	54.1 ± 13.5	0.16	21.8 ± 8.4	22.1 ± 8.0	0.67
Demographics							
Age	49.9 ± 6.9	48.5 ± 5.9	52.3 ± 7.8	**<0.001**	---	---	---
Race/ethnicity				**0.001^**			**<0.001^**
NH Black	204 (28.5)	148 (32.2)	56 (21.7)		140 (36.8)	64 (19.0)	
NH White	451 (62.9)	266 (58.0)	185 (71.7)		188 (49.5)	263 (78.0)	
Hispanic	56 (7.8)	39 (8.5)	17 (6.6)		47 (12.4)	9 (2.7)	
Other	6 (0.8)	6 (1.3)	0 (0.0)		5 (1.3)	1 (0.3)	
Education				**<0.001**			**<0.001**
<HS	46 (6.4)	36 (7.8)	10 (3.9)		38 (10.0)	8 (2.4)	
HS graduate	93 (13.0)	64 (13.9)	29 (11.2)		64 (16.8)	29 (8.6)	
Some college	189 (26.36)	140 (30.5)	49 (19.0)		114 (30.0)	75 (22.3)	
College graduate	176 (24.6)	105 (22.9)	71 (27.5)		98 (25.8)	78 (23.2)	
Beyond college graduate	213 (29.7)	114 (24.8)	99 (38.4)		66 (17.4)	147 (43.6)	
Income, <$30,000/year	313 (43.8)	222 (48.4)	91 (35.6)	**<0.001**	201 (52.9)	112 (33.4)	**<0.001**
Behavioral risk factors							
Smoking status				**0.003**			**<0.001**
Never	210 (29.4)	127 (27.9)	83 (32.2)		111 (29.4)	99 (29.5)	
Former	288 (40.3)	171 (37.5)	117 (45.4)		120 (31.8)	168 (50.0)	

Current	216 (30.3)	158 (34.7)	58 (22.5)		147 (38.9)	69 (20.5)	
Alcohol use				0.07			**0.04**
None	122 (17.1)	90 (19.7)	32 (12.4)		74 (19.6)	48 (14.3)	
Low – moderate	444 (62.2)	271 (59.4)	173 (67.1)		223 (59.0)	221 (65.8)	
Moderate – heavy	96 (13.5)	60 (13.2)	36 (14.0)		47 (12.4)	49 (14.6)	
Binge	52 (7.3)	35 (7.7)	17 (6.6)		34 (9.0)	18 (5.4)	
Cardiometabolic risk factors							
BMI	25.9 ± 4.6	25.6 ± 4.2	26.5 ± 5.1	**0.02**	25.9 ± 4.7	25.9 ± 4.4	0.91
Systolic BP	124.0 ± 12.6	123.7 ± 12.5	124.7 ± 12.7	0.33	123.8 ± 12.0	124.3 ± 13.3	0.58
Diastolic BP	74.8 ± 8.5	74.8 ± 8.5	74.7 ± 8.4	0.88	74.7 ± 8.7	74.9 ± 8.2	0.75
Hypertension mediation usage	165 (23.0)	101 (22.1)	64 (24.8)	0.40	58 (15.3)	107 (31.8)	**<0.001**
Diabetes	71 (10.6)	46 (10.6)	25 (10.4)	0.93	30 (8.3)	41 (13.1)	**0.04**
Total cholesterol	195.5 ± 42.1	192.8 ± 44.2	200.2 ± 37.6	**0.02**	190.4 ± 41.3	201.2 ± 42.2	**<0.001**
HDL cholesterol	47.0 ± 13.9	45.4 ± 14.2	50.0 ± 12.9	**<0.001**	46.3 ± 14.3	47.9 ± 13.4	0.12
Cholesterol medication usage	172 (24.1)	126 (27.6)	46 (17.8)	**0.003**	69 (18.3)	103 (30.7)	**<0.001**
Dyslipidemia	561 (81.3)	380 (85.6)	181 (73.6)	**<0.001**	289 (79.0)	272 (84.0)	0.09
eGFR	87.3 ± 17.7	89.4 ± 17.9	83.5 ± 16.6	**<0.001**	92.6 ± 17.4	81.2 ± 16.0	**<0.001**
Creatinine	1.1 ± 0.2	1.1 ± 0.2	1.1 ± 0.2	0.25	1.0 ± 0.2	1.1 ± 0.2	**0.04**
HIV-related factors							
HIV positive	459 (64.0)	---	---	---	276 (72.6)	183 (54.3)	**<0.001**
CD4 count	550.9 ± 279.0	---	---	---	531.2 ± 271.2	580.8 ± 288.8	0.06
Nadir CD4	298.5 ± 199.1	---	---	---	311.1 ± 209.5	280.0 ± 181.8	0.10
Undetectable viral load	284 (61.9)	---	---	---	161 (58.3)	123 (67.2)	0.06
Duration of ART	4.7 ± 3.1	---	---	---	4.5 ± 3.1	4.8 ± 3.1	0.29
Type of ART		---	---	---			0.85^
None	120 (26.3)				74 (27.0)	46 (25.3)	
PI	160 (35.1)				97 (35.4)	63 (34.6)	
NNRTI	159 (34.9)				91 (33.2)	68 (37.4)	
NRTI	16 (3.5)				11 (4.0)	5 (2.8)	
Other	1 (0.2)				1 (0.4)	0 (0.0)	
History of AIDS/other opportunistic infections	67 (14.6)	---	---	---	37 (13.4)	30 (16.4)	0.37

Continuous variables are reported as mean (SD); categorical variables are reported as n (%). Differences in continuous variables were assessed using one-way ANOVA (sum of squares). Differences in categorical variables were assessed using Chi-square tests or Fisher's exact tests (marked with ^) when cell sizes were small. HIV-related factors are presented only for men with HIV (MWH). Abbreviations include: MWH, men with HIV; MWoH, men without HIV; ART, antiretroviral therapy; PI, protease inhibitor; NNRTI, non-nucleoside reverse transcriptase inhibitor; NRTI, nucleoside reverse transcriptase inhibitor; BMI, body mass index; BP, blood pressure; HDL, high-density lipoprotein; eGFR, estimated glomerular filtration rate; CD4, CD4+ T lymphocyte count; AIDS, acquired immunodeficiency syndrome

Supplemental Figure 1.

Figure. Adjusted longitudinal changes in cognitive function in relation to tertiles of carotid distensibility stratified by HIV serostatus

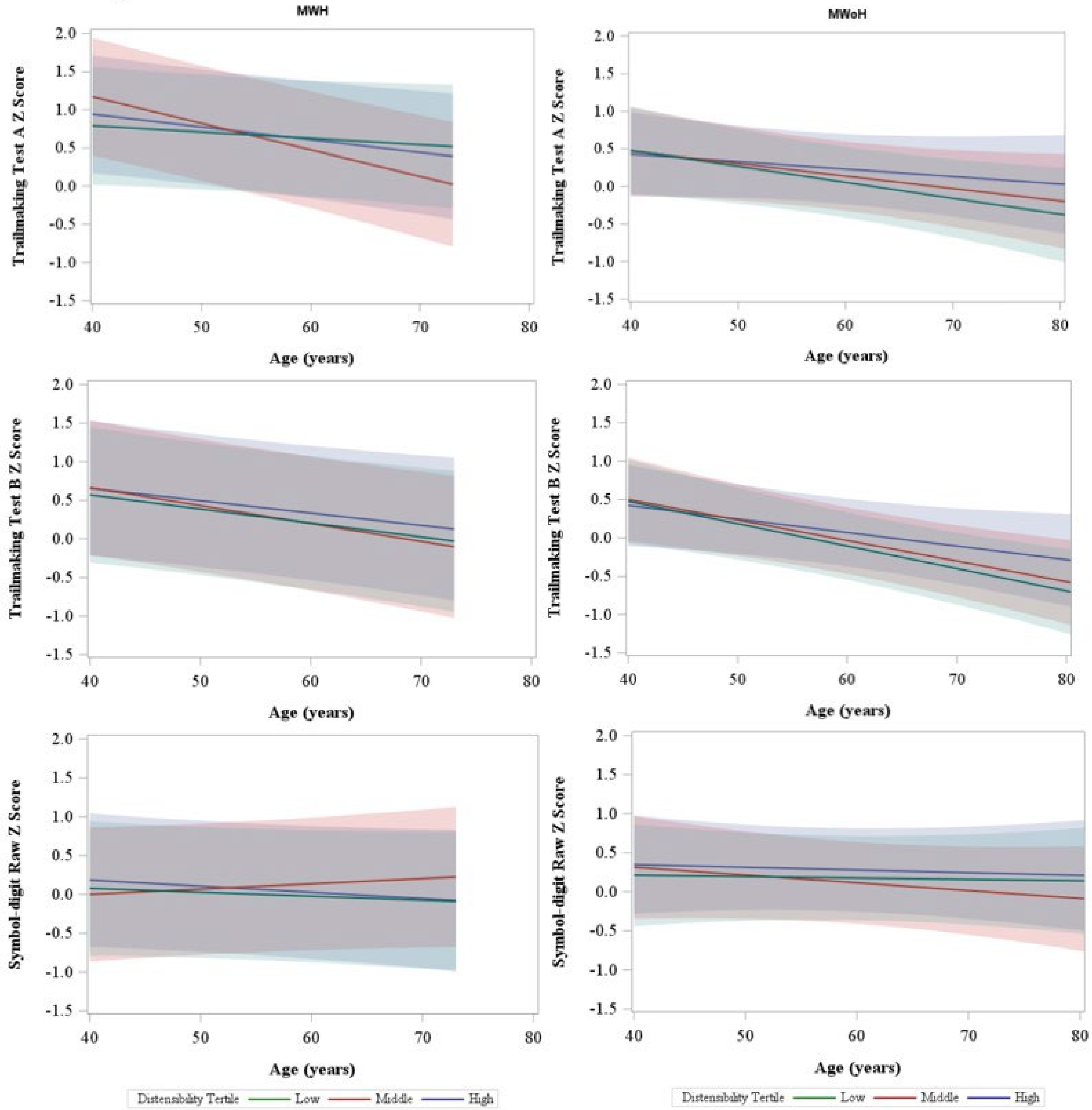

Supplement: Supplementary file 1 [file glag220_supplementary_data.pdf]
